# Weld Line Strength of Polyamide Fiberglass Composite at Different Processing Parameters in Injection Molding Technique

**DOI:** 10.3390/polym15204102

**Published:** 2023-10-16

**Authors:** Van-Thuc Nguyen, Tran Minh The Uyen, Pham Son Minh, Thanh Trung Do, Trung H. Huynh, Tronghieu Nguyen, Vinh Tien Nguyen, Van Thanh Tien Nguyen

**Affiliations:** 1 Faculty of Mechanical Engineering, Ho Chi Minh City University of Technology and Education, Ho Chi Minh City 71307, Vietnam; nvthuc@hcmute.edu.vn (V.-T.N.); uyentmt@hcmute.edu.vn (T.M.T.U.); minhps@hcmute.edu.vn (P.S.M.);; 2 Faculty of Mechanical Engineering, Industrial University of Ho Chi Minh City, Nguyen Van Bao Street, Ward 4, Go Vap District, Ho Chi Minh City 70000, Vietnam

**Keywords:** filling time, packing time, tensile strength, mold temperature, ANNs

## Abstract

This study examines the impact of injection parameters on the weld line strength of the polyamide 6 and 30% fiberglass (PA6 + 30% FG) composite samples. The effects of filling time, packing time, packing pressure, melt temperature, and mold temperature on the ultimate tensile strength (UTS) and the elongation value of the weld line are investigated. The results reveal that the filling time factor has the lowest influence rate. On the contrary, the packing pressure has the most considerable value of UTS standard deviation, indicating that this factor has a high impact rate. The melt temperature factor has the highest elongation standard deviation, pointing out the strong impact of melt temperature on the elongation value. In reverse, the filling time factor has the lowest elongation standard deviation, showing the low impact of this factor on the elongation value. Increasing the mold temperature enhances the elongation value greatly because a higher temperature generates a better connection in the weld line area. Although the UTS value improves modestly when the mold temperature control system is used, the elongation result from the mold temperature parameter is better than expected. The UTS result from all parameters presents a minor deviation; therefore, it is lower than expected. The optimal strength result from artificial neural networks with genetic algorithm optimization is 85.1 MPa, which is higher than the best experiment result of 76.8 MPa. Scanning electron microscopy (SEM) results show that the interface between the fiberglass and the PA matrix has high adherence. The fracture surface is smooth, indicating that the PA6 + 30% FG composite sample has a high fragility level. The findings could help to increase the injection sample’s weld line strength by optimizing the injection molding conditions.

## 1. Introduction

Molten polymers gradually lose temperature during the injection molding process as heat is transmitted to the mold body. The skin layer of the solid polymer is able to reduce the flow of the molten polymer [1,2,3]. In many circumstances, this undesirable quick solidification process may result in incomplete cavity filling and loss of filling pressure. As a result, the quality of injectable products falls. In order to solve this issue, mold temperature control is typically used to impede the development of a thick skin layer. The mold temperature is controlled by a heat-assisted system [4,5,6]. The heat source could be gas, steam, or high-frequency-induced heating. Many investigations tried to uncover the impacts of mold temperature on the characteristics of injection samples. Wang et al. [7], for example, used a steam heating system to adjust the mold temperature during the injection molding process. Yan et al. [8] used gas-assisted mold temperature control and found that the mold temperature has a greater influence on cooling rate control than the melt temperature.

When high strength, high elasticity, high temperature resistance, and high abrasion resistance are required, polyamide (PA) polymers are frequently chosen [9,10,11]. PA are used in a variety of industries, including those related to housing, solar energy, sports and recreation, and electronic engineering [12,13,14]. Significantly, PAs’ mechanical qualities can be significantly enhanced when strengthened with certain fillers such as fiberglass, carbon fiber, and talc powder [15,16,17]. The composite’s fiber enhancement mechanism is the transportation of the applied stress from the matrix to the fibers [18]. Peng et al. [19] mixed PA with carbon fiber to conduct a fused deposition modeling technique. The printing parameters, such as feed rate, layer thickness, and orientation on the tensile strength, are investigated. Fiberglass is a common thermoplastic polymer-reinforcing component. It can be used with PA to reinforce the polymer matrix. Eriksson et al. [20], for example, investigated the effects of fiber shortening on the strength of a PA composite. The tensile strength of the PA composite increases as fiber length increases. Pinto et al. [21] studied the effects of ionizing radiation on PA6 with 30% fiberglass and discovered that the irradiation method might increase the tensile strength of the composite by improving cross-linking. Moreover, Jeun et al. [22] irradiated PA12 composite mixed with fiberglass by an electron beam. The irradiated PA12 composite has higher wear resistance due to the scission of molecules and chain folds. Reinforcing with fiberglass and aramid fabric with a laminar structure creates a sandwich-form composite with a super high impact toughness [23]. This composite could produce ballistic protection panels with an urban armor level III-A. The effects of the fiber orientation distribution on the mechanical properties of the PA6 reinforced with 30% fiberglass have been reported by A. Bernasconi et al. [24]. According to the findings, the composite’s elastic modulus, tensile strength, and fatigue strength all dropped as the orientation angle was increased.

The molten plastic is gradually cooled down during the injection process in the injection molding technique. When two plastic streams meet, they can generate a weld line. The formation of the weld line reduces the strength of the injection parts. However, multiple techniques, such as adding more reinforcement, modifying the structure, or altering the injection parameters, can reduce the impact of weld lines. Wu et al. [25] studied the influence of process factors on the mechanical characteristics of PP and HDPE, for example. The study revealed that the melt temperature has a more substantial influence than the packing time and packing pressure on the weld line strength of the injected samples. Li et al. [26] reported that the study could use the properties of the molecular backbone orientation to determine the location of the weld line. According to Ersoy et al. [27], talc filler could improve the weld line strength of PA polymer. Surprisingly, Kagitci et al. [28] showed that increasing the injection pressure increased the weld line strength in the PA6 composite reinforced with short fiberglass. This study, however, was limited to a single unique shape portion.

Weld line effect could be limited by applying techniques such as improving the mold design, optimizing the molding parameters, or using air-venting. Koponen et al. [29] suggested that improving the flow rate could increase the weld line strength of the injected molding sample. Improving mold design by optimizing the gate location might also strengthen the weld line area, according to Munankar et al. [30]. Moreover, an optimized clamping force could prevent the formation of weld lines by improving the air-venting performance [31,32]. Interestingly, adding a compatibilizer could promote better polymer morphology and improve weld line strength [33]. Michaeli et al. [34] prevented the formation of weld lines by preheating before injection and cooling before demolding. On the other hand, Liu et al. [35] focused on optimizing the sample’s geometry shape to improve the weld line strength.

The injection parameters can be easily controlled under a panel controller system in the injection technique. Therefore, optimizing the injection process by examining many parameters is necessary for a specific molding condition: for example, the PA6 and fiberglass composite. In addition, the effects of a heat-assisted mold temperature control system on the quality of the weld line also need more consideration, especially in relation to other parameters. Previously, other studies mostly focused on a single parameter. The effects of multi-parameters are not thoroughly investigated. Specifically, the impacts of mold temperature with mold temperature control, melt temperature, filling time, packing time, and packing pressure on the characteristics of PA6 mixed with 30% short fiberglass (PA6 30% FG) are rarely discussed and need more investigation.

This study examines the impacts of multiple parameters such as melt temperature, filling time, packing time, and packing pressure on the mechanical properties of PA6 30% FG. Especially, the mold temperature effect with a gas-assisted mold temperature control system is also mentioned in this report. The fractured sample is also analyzed via SEM microstructure. The experimental results are optimized by using artificial neural network (ANN) methods. The findings might demonstrate which parameters have the most influence on the mechanical properties of the weld line area, as well as the efficiency of the mold temperature control system in improving the injection sample’s weld line strength.

## 2. Experimental Methods

**Figure 1:**
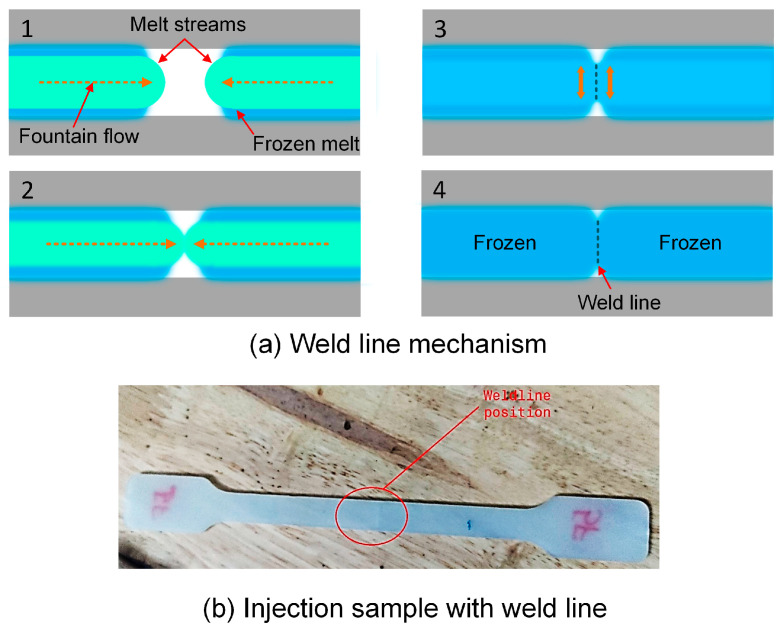
The weld line formation mechanism and injection sample: (**a**) weld line mechanism, and (**b**) injection sample with weld line.

**Figure 2:**
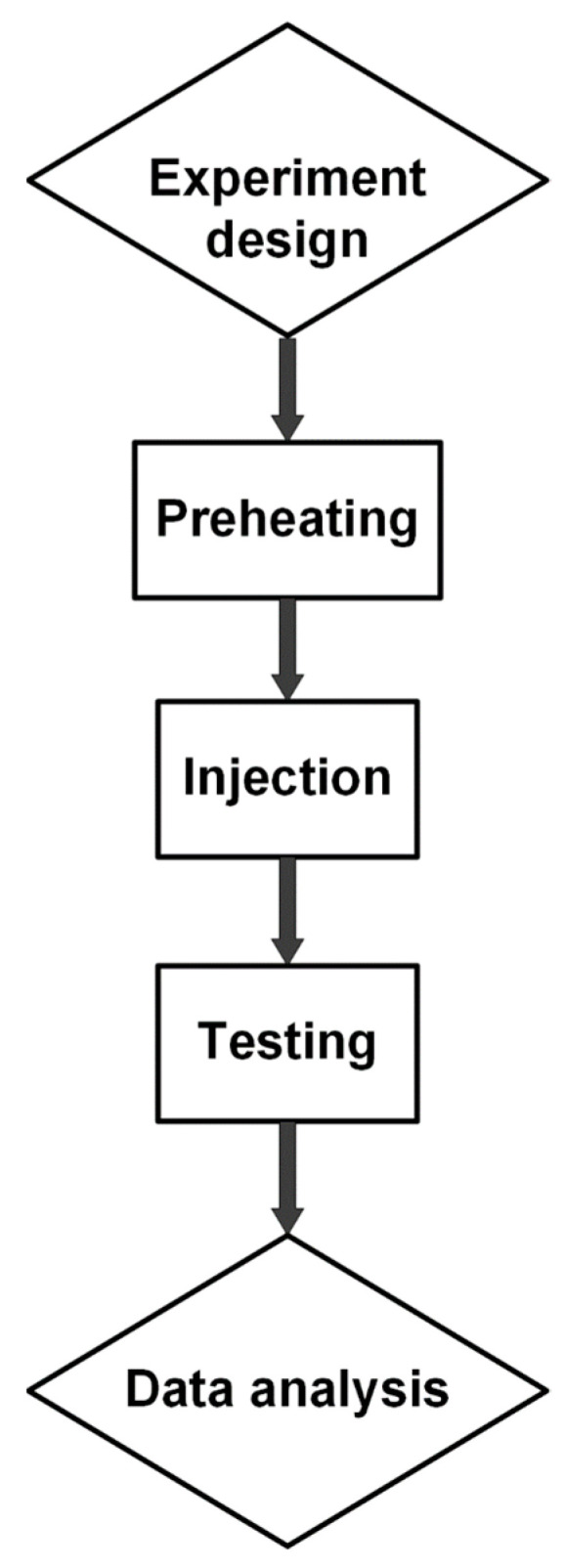
Summary diagram of the experimental process.

Figure 1a shows the weld line formation mechanism. The sample shape is designed based on ASTM D638 standards, as shown in Figure 1b [36]. The experimental process is presented in Figure 2. Before injection, PA6 polymer with 30% FG from Akulon^®^ K224-G6, DSM Company, Heerlen city, The Netherlands, is dried at 85 °C for 12 h to remove the humidity. The polymer has a UTS of 110 MPa, an elongation value of 7%, and a melt temperature for injection molding of 270–290 °C. The injection molding machine type used in this study is MA 1200III (Haitian, China). This is a screw injection molding machine with a screw diameter of 36 mm, injection capacity of 157 g, screw speed of 122 mm/s, a nozzle of 3 mm with a temperature limit switch control, and a clamping force of 1200 kN. The pressure, temperature, and other filling parameters are controlled by using a system of pressure, temperature, and displacement sensors under the controller PILOT5 system named Haitian Techmation Tech2 controller with a panel. These parameters are modified by using this type of control panel. To investigate the injection parameters, the filling time, packing time, packing pressure, and melt temperate are examined, as presented in Table 1. In the first group with samples 1–5, the filling times vary from 3.0 s to 3.8 s, while the packing time is fixed at 0.4 s, the packing pressure is 59 MPa, and the melt temperature is 269 °C. This group examines the effects of filling time on the properties of the sample. Similarly, the second group with samples 6–10 surveys the effect of packing time in the range of 0–0.8 s. The third group with samples 11–15 surveys the impact of packing pressure from 55 MPa to 63 MPa. Finally, the fourth group with samples 16–20 investigates the effect of melt temperature on the sample properties by modifying it in the range of 265–273 °C. There are five samples for each experiment number, repeating each molding condition. The tensile strength and elongation values are present in an average number with error bars. The error bars in the result diagrams present the deviation of the mechanical value around an average number.

There are limiting processing conditions, depending on the type of machine and materials. For example, with PA6 polymer with 30% FG from Akulon^®^ K224-G6, the melt temperature range is 260–275 °C. Therefore, we investigated the effects of melt temperature in the range of 265–273 °C. Moreover, with the injection molding machine named MA 1200III (Haitian, China), the other injection parameters are set according to the technical manual guide. For instance, the filling time refers to the time necessary from the start of injection molding to the filling of the mold chamber. The filling time has a direct impact on the quality and appearance of the molded parts. The following criteria should be considered while determining the filling time: the injection molding machine’s injection capacity and maximum flow rate; the mold’s structure and size; plastic material properties such as viscosity, melting temperature, and so forth; and filling speed, filling pressure, and other injection molding process parameters. The lower filling time can lead to insufficient melt plastic, therefore causing the short shot phenomenon. The maximum condition until degradation of the material starts is melt temperature rather than time. With PA6 and fiberglass material, the maximum temperature is over 290 °C; therefore, the melt plastic is not easy to degrade under 265–273 °C conditions. Furthermore, the maximum time for degradation under this temperature range is 4–5 h, which is significantly longer than the injection time.

The injection samples are tested by a tensile test machine AG-X Plus 20 kN (Shimadzu, Japan) at a 5 mm·min^−1^ speed and a grips distance of 135 mm, following ASTM D638 standards [36]. After the tensile test, the fracture surfaces are examined by scanning electron microscope (SEM) TM4000 (Hitachi, Japan).

## 3. Results and Discussion

### 3.1. Effects of Injection Parameters

**Figure 3:**
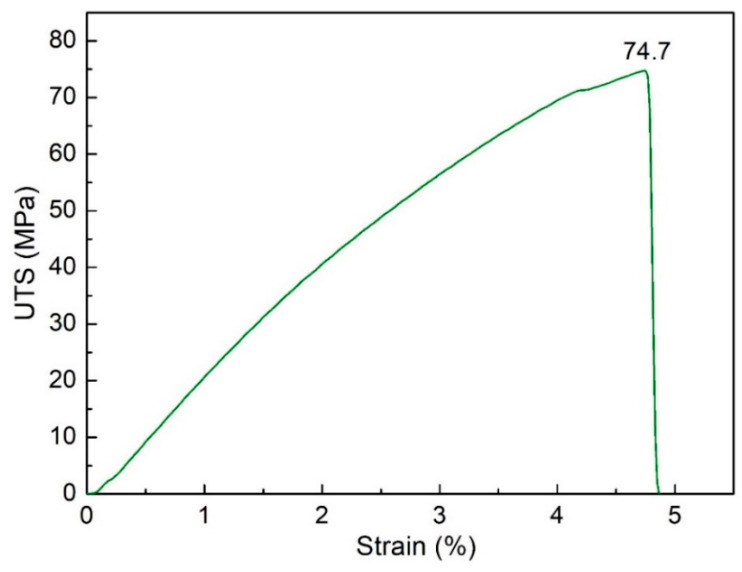
Stress-strain diagrams of sample 1 of PA6 + 30% FG composite.

**Figure 4:**
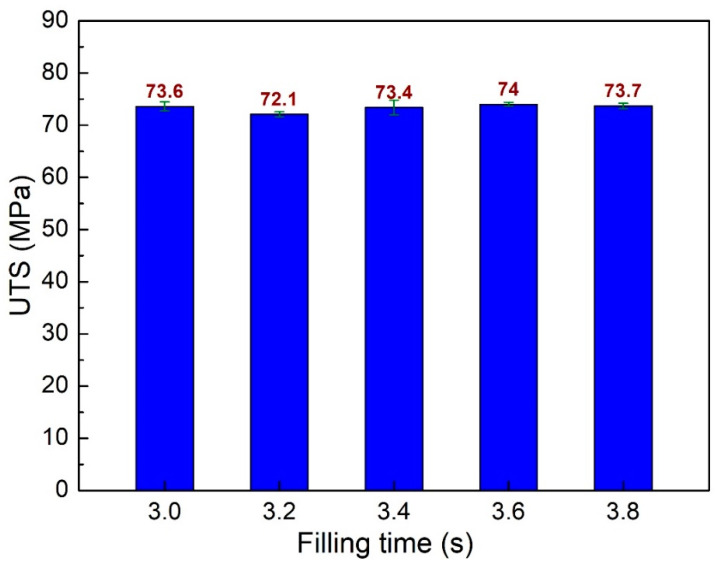
Comparison of UTS values of PA6 + 30% FG composite samples with weld lines at different filling times.

Firstly, the impacts of filling time are examined. The filling time is surveyed from 3.0 s to 3.8 s. Figure 3 shows the stress-strain curve of sample 1 of PA6 + 30% FG composite samples with a weld line. The sample elongation is less than 5%, indicating the low ductility of the weld line area [25,37,38]. Figure 4 compares ultimate tensile strength (UTS) values of PA6 + 30% FG composite samples with weld lines at different filling times. The UTS values are 73.6 MPa, 72.1 MPa, 73.4 MPa, 74 MPa, and 73.7 MPa, corresponding to 3.0 s, 3.2 s, 3.4 s, 3.6 s, and 3.8 s. The average UTS value is 73.36 MPa, with a low standard deviation of 0.7 MPa. As mentioned in Section 2, this UTS value is lower than the PA6 + 30% FG composite samples without a weld line, indicating that the weld line negatively impacts the UTS value [39,40,41]. Because the weld line decreases the continuity of the injection sample, it reduces the UTS value of the sample. The UTS value reaches the highest value of 74 MPa at 3.6 s, while the lowest UTS value is 72.1 MPa at 3.2 s. This result indicates that the difference in the UTS values between these samples is relatively low. The filling time from 3.0 s to 3.8 s is sufficient for the injection process. In other words, with the filling time from 3.0 s to 3.8 s, the UTS value only oscillates in a short range, from 72.1 MPa to 74 MPa.

**Figure 5:**
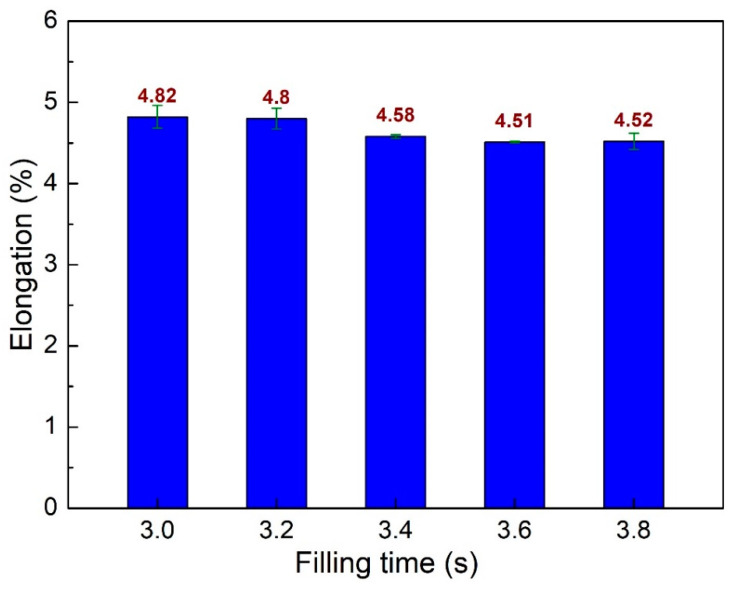
Comparison of elongation values of PA6 + 30% FG composite samples with weld lines at different filling times.

Figure 5 compares elongation values of PA6 + 30% FG composite samples at different filling times. The elongation values are 4.82%, 4.8%, 4.58%, 4.51%, and 4.52% corresponding to 3.0 s, 3.2 s, 3.4 s, 3.6 s, and 3.8 s. In general, improving the filling time leads to a slight reduction in the sample elongation, a consistent result compared to the report of Cox et al. [42]. These values are lower than the sample elongation without the weld line, which is 7%, as shown in Section 2. This phenomenon pinpoints the reduction of the ductility in the weld line area due to the discontinuity of the sample structure [39]. Moreover, similar to the low oscillation of UTS values, the average elongation value is 4.65%, with a low standard deviation of 0.14%. It means that the filling time also has a low influence on the elongation value, with the highest elongation of 4.82% at 3.0 s and the lowest one at 3.6 s. On the contrary, the highest UTS value of 74 MPa is obtained at 3.6 s.

**Figure 6:**
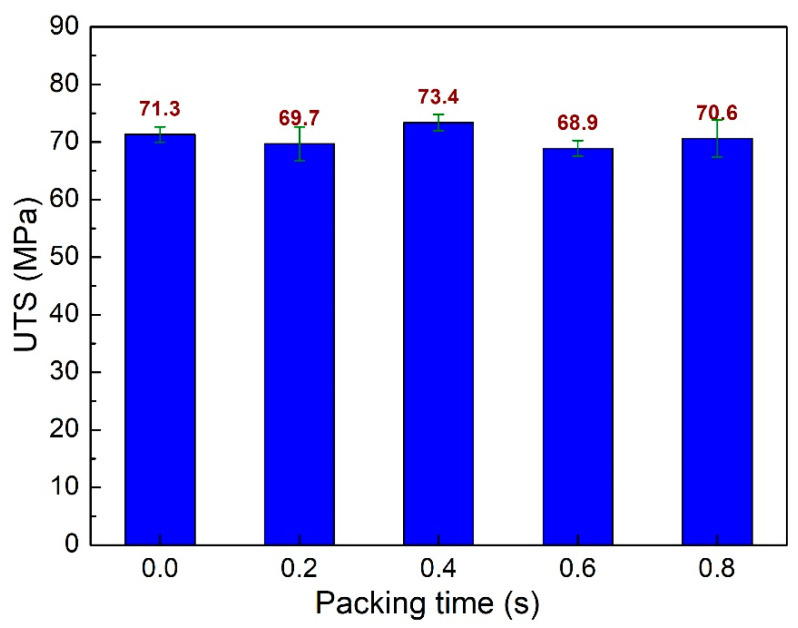
Comparison of UTS values of PA6 + 30% FG composite samples with weld lines at different packing times.

Packing time is an essential parameter in the injection molding process. Applying the packing step could remove more air bubbles in the sample. Therefore, the quality of the injection sample could be improved. This study investigates the packing time from 0.0 s to 0.8 s. Figure 6 shows the UTS values of PA6 + 30% FG composite samples with weld lines at different packing times. The UTS values are 71.3 MPa, 69.7 MPa, 73.4 MPa, 68.9 MPa, and 70.6 MPa, corresponding to 0 s, 0.2 s, 0.4 s, 0.6 s, and 0.8 s. The average UTS value for all cases is 70.8 MPa, with a standard deviation value of 1.54 MPa. Packing at 0.4 s has a higher UTS value than without the packing step. However, packing at 0.2 s, 0.6 s, and 0.8 s results in a lower UTS value. The optimal packing time for the highest UTS value is 0.4 s.

**Figure 7:**
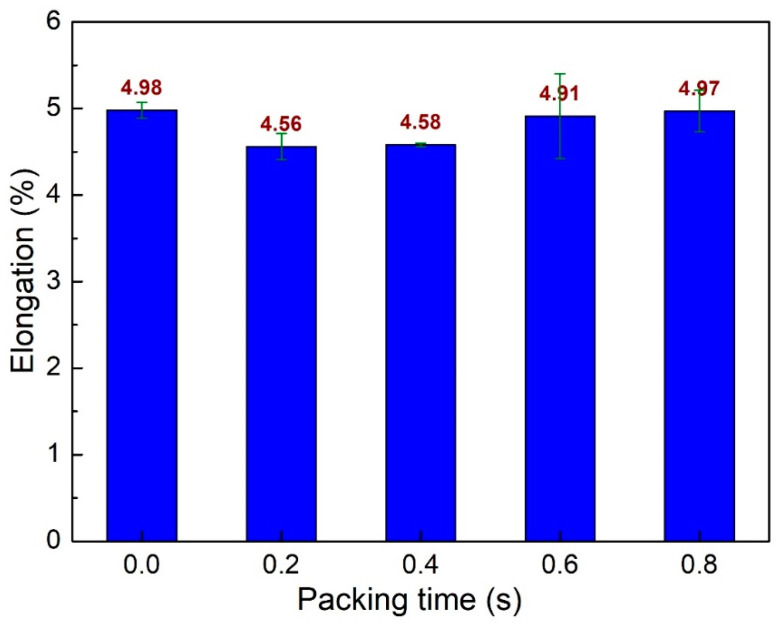
Comparison of elongation values of PA6 + 30% FG composite samples with weld lines at different packing times.

Figure 7 shows the elongation values of PA6 + 30% FG composite samples with weld lines at different packing times. The elongation values are 4.98%, 4.56%, 4.58%, 4.91%, and 4.97% corresponding to 0 s, 0.2 s, 0.4 s, 0.6 s, and 0.8 s. The average elongation value is 4.8%, with a standard deviation value of 0.19%, indicating the stability of the elongation value when changing the packing time. Samples have packing times at 0 s, 0.6 s, and 0.8 s and have higher elongation values than that of 0.2 s and 0.4 s; however, the difference is slight.

**Figure 8:**
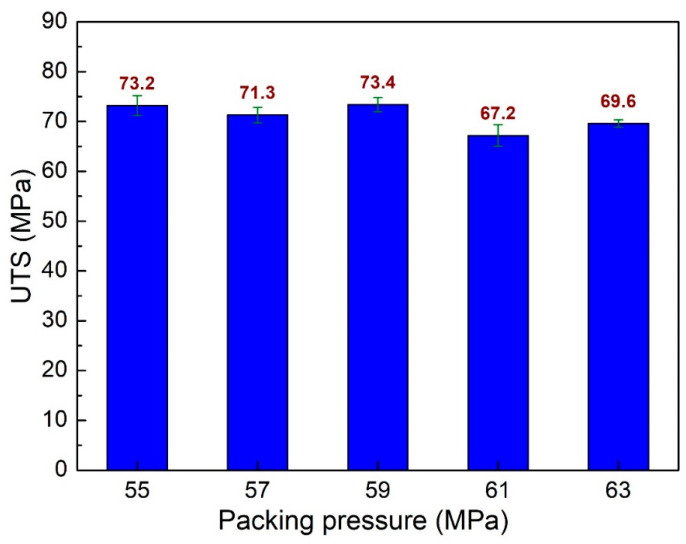
Comparison of UTS values of PA6 + 30% FG composite samples with weld lines at different packing pressures.

Separate from the packing time parameters, packing pressure also impacts the weld line characteristics. Figure 8 shows the UTS values of PA6 + 30% FG composite samples with weld lines at different packing pressures. The UTS values are 73.2 MPa, 71.3 MPa, 73.4 MPa, 67.2 MPa, and 69.6 MPa, corresponding to the packing pressure of 55 MPa, 57 MPa, 59 MPa, 61 MPa, and 63 MPa. The average UTS value for all cases is 70.94 MPa, with a standard deviation of 2.33 MPa. The packing pressure has a higher effect with a higher standard deviation value than the packing time cases. The average UTS value of the packing pressure case is 70.94 MPa, which is also higher than the packing time cases with 0.8 MPa. The PA6 + 30% FG composite sample with a packing pressure of 59 MPa has the highest UTS value, 73.4 MPa, while the lowest one is 67.2 MPa, corresponding to the packing pressure of 61 MPa.

**Figure 9:**
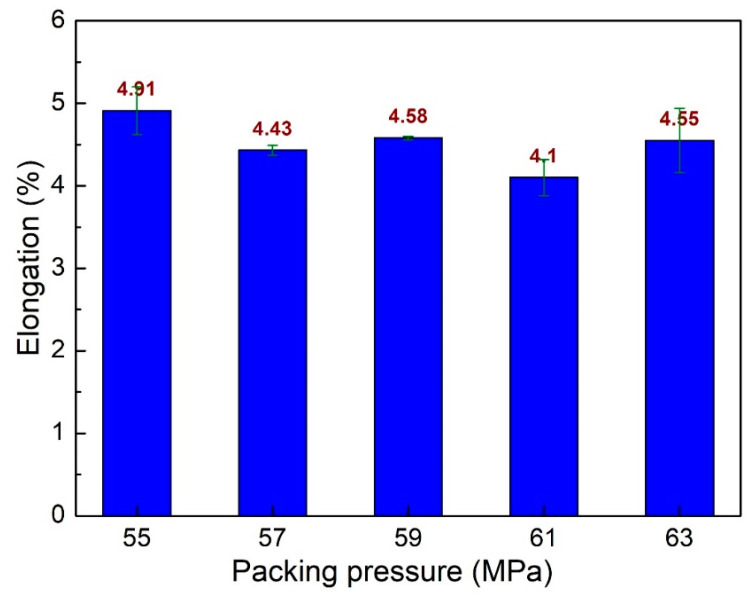
Comparison of elongation values of PA6 + 30% FG composite samples with weld lines at different packing pressures.

Figure 9 shows the elongation values of PA6 + 30% FG composite samples with weld lines at different packing pressures. The elongation values are 4.91%, 4.43%, 4.58%, 4.1%, and 4.55%, corresponding to the packing pressures of 55 MPa, 57 MPa, 59 MPa, 61 MPa, and 63 MPa. The average elongation value is 4.51%, with a standard deviation value of 0.23%. Notably, the average elongation of these packing pressure cases is smaller than the packing time and filling time cases.

**Figure 10:**
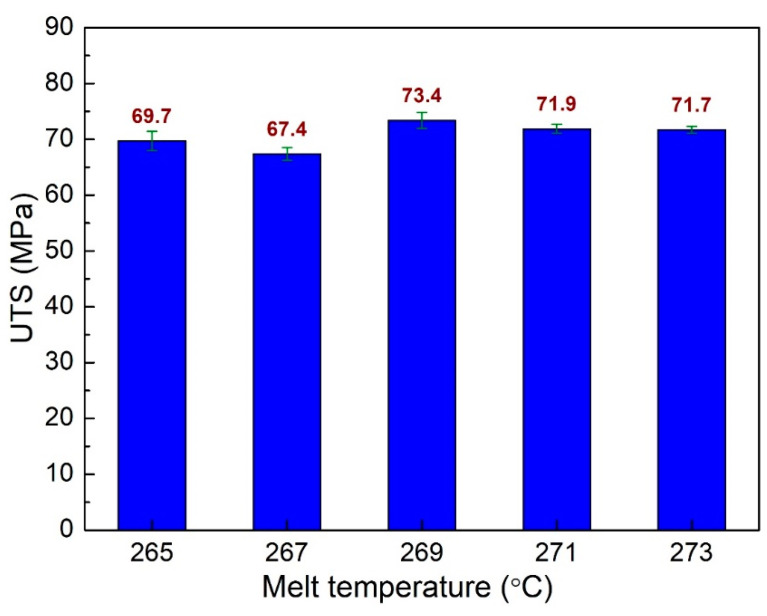
Comparison of UTS values of PA6 + 30% FG composite samples with weld lines at different melt temperatures.

Melt temperature is an essential factor that strongly affects the injection molding process. A low melt temperature could lead to high viscosity and hinders the filling process. However, a high melt temperature could result in a degradation of the polymer. Therefore, the quality of the injection samples is negatively affected. This report surveys the impacts of melt temperature on the mechanical properties of the PA6 + 30% FG composite samples. The melt temperature ranges from 265 °C to 273 °C. Figure 10 demonstrates the UTS values of PA6 + 30% FG composite samples with weld lines at different melt temperatures. The UTS values are 69.7 MPa, 67.4 MPa, 73.4 MPa, 71.9 MPa, and 71.7 MPa corresponding to 265 °C, 267 °C, 269 °C, 271 °C, and 273 °C. The average UTS value for all cases is 70.82 MPa, with a standard deviation value of 2.08 MPa. The highest UTS value of 73.4 MPa is obtained at 269 °C, while at 267 °C, the PA6 + 30% FG composite sample has the lowest UTS value of 67.4 MPa. Moreover, the higher melt temperature sample group at 269 °C, 271 °C, and 273 °C has higher UTS values than the lower group at 265 °C and 271 °C melt temperatures. The reason could be the more elevated temperature leading to a smoother flow rate, resulting in a higher weld line quality, a consistent result as shown in a study by Kitayama et al. [43].

**Figure 11:**
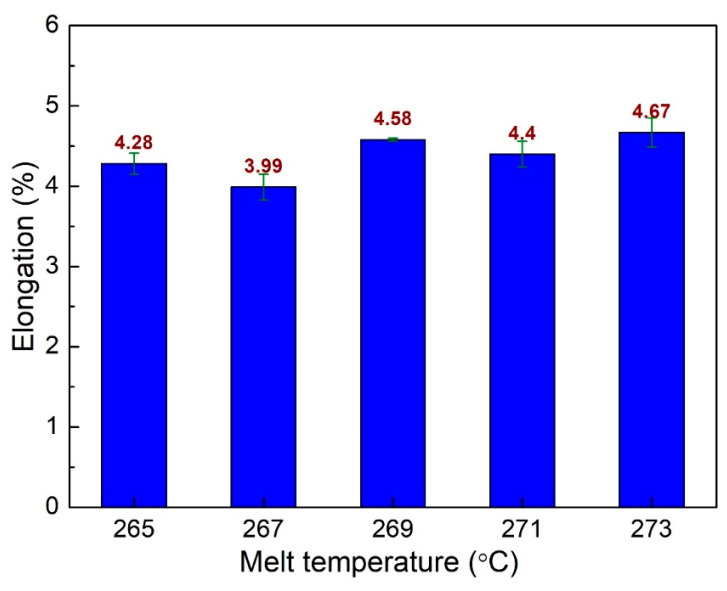
Comparison of elongation values of PA6 + 30% FG composite samples with weld lines at different melt temperatures.

Figure 11 shows the elongation values of PA6 + 30% FG composite samples with weld lines at different melt temperatures. The elongation values are 4.28%, 3.99%, 4.58%, 4.4%, and 4.67% corresponding to 265 °C, 267 °C, 269 °C, 271 °C, and 273 °C. The average elongation value is 4.30%, with a standard deviation value of 0.39%. The highest elongation value is gained at 273 °C, while the lowest appears at 267 °C. Compared to the samples when surveying the effects of filling time, packing time, and packing pressure, these cases have a lower elongation value. Generally, the highest UTS value of 73.7 MPa is achieved by sample 5 with the injection parameters of a filling time of 3.8 s, packing time of 0.4 s, packing pressure of 59 MPa, and a melt temperature of 269 °C. Sample 6 has the highest elongation value, which is 4.98%. The injection parameters of sample 6 are: a filling time of 3.4 s, a packing time of 0 s, packing pressure of 59 MPa, and a melt temperature of 269 °C.

Previous studies mostly concentrate on optimizing a specific parameter. This study examines a wider range of parameters and try to indicate the effective rate of each parameter. Therefore, selecting an important parameter for improving the quality of weld lines can become easier. Table 2 presents the average UTS and elongation values with standard deviations of the PA6 + 30% FG composite samples with weld lines of different injection factors. The filling time factor has the highest average UTS value compared to other factors, indicating that we could modify the filling time in the mentioned range while preserving the high value of UTS. Moreover, the standard deviation of the UTS value of the filling time factor is 0.70, which is the lowest value, showing the low impact rate of this factor compared to the other elements. In other words, changing the filling time in this range results in low variation in the UTS value. On the contrary, the packing pressure has the highest UTS standard deviation value, indicating this factor’s high impact rate.

The packing time factors have the highest average elongation values of 4.80%. The melt temperature factor has the lowest average elongation value of 4.30%. Separately, it also has the highest elongation standard deviation of 0.39%, pointing out the strong impact of melt temperature on the elongation value. The higher temperature leads to a smoother flow rate, resulting in a higher weld line quality [43]. In reverse, the filling time factor has the lowest elongation standard deviation of 0.14%, showing the low effect of this factor on the elongation value. Compared to the filling time factor, the packing pressure with an elongation standard deviation value of 0.23% has a higher rate of influence. A higher injection pressure could improve weld line strength in the PA6 composite reinforced with short fiberglass [28]. In summary, to improve the UTS value of the PA6 composite, the packing pressure factor must be carefully considered. The melt temperature factor must be noticed when considering the elongation value.

**Figure 12:**
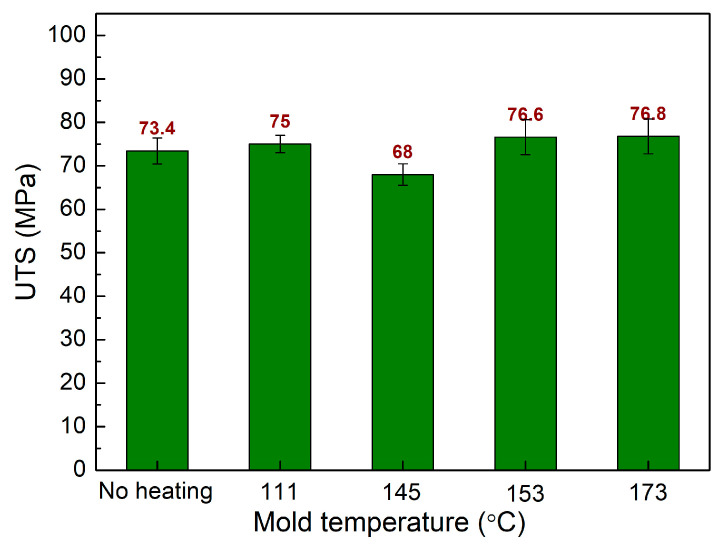
Comparison of UTS values of PA6 + 30% FG composite samples with weld lines at different mold temperatures.

As well as optimizing the injection parameters that are controlled by the injection machine, this study also conducted a gas-assisted mold temperature control system. The mold is heated by a Makita HG6530V heat gun on the weld line area at different times before injection molding. This mold temperature control system is a simple system that can be set up easily with an affordable cost, requiring mainly a heat gun. To improve the filling rate, raising the mold temperature with a heat gun is an effective method. The higher mold temperature facilitates the filling process; therefore, the injection sample quality is improved. This study examines the influences of mold temperature ranging from room temperature to 173 °C, a filling time of 3.4 s, a packing time of 0.4 s, a packing pressure of 59 MPa, and a melt temperature of 269 °C. Figure 12 shows the comparison of UTS values of PA6 + 30% FG composite samples with weld lines at different mold temperatures. The UTS values are 73.4 MPa, 75 MPa, 68 MPa, 76.6 MPa, and 76.8 MPa corresponding to room temperature, 111 °C, 145 °C, 153 °C, and 173 °C. Increasing the mold temperature mostly leads to a slight improvement in the UTS value. The reason for this enhancement is the better connection in the weld line area at a higher temperature [37,38]. However, at 145 °C, the UTS value is lower than samples without heating. In general, increasing the mold temperature to 111 °C, 153 °C, and 173 °C results in a higher level of UTS value. The improvement is not significant and can be explained by the existence of a bridging mechanism of the fiberglass mentioned in the next section.

**Figure 13:**
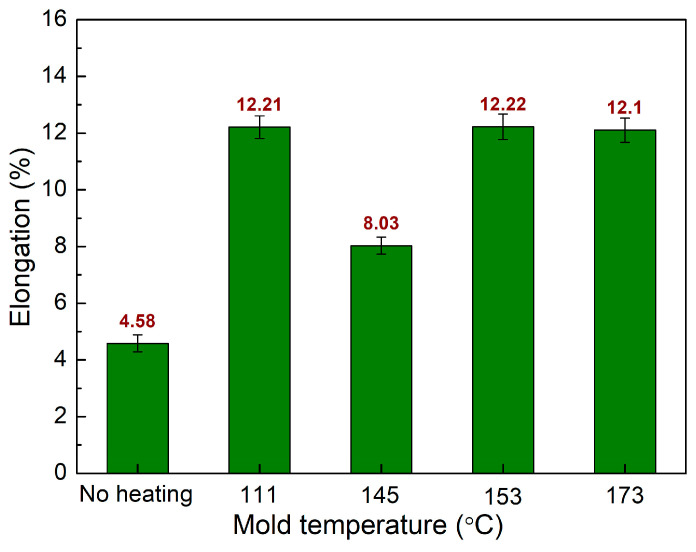
Comparison of elongation values of PA6 + 30% FG composite samples with weld lines at different mold temperatures.

The elongation values of PA6 + 30% FG composite samples with weld lines at various melt temperatures are shown in Figure 13. The elongation values are 4.58%, 12.21%, 8.03%, 12.22%, and 12.1% corresponding to room temperature, 111 °C, 145 °C, 153 °C, and 173 °C. Remarkably, increasing the mold temperature significantly improves the elongation value due to the fact that a higher temperature produces a better connection in the weld line area [44,45]. The highest elongation value of 12.22% is obtained when heating the mold to 153 °C. In summary, increasing the mold temperature to 153 °C and 173 °C leads to the better UTS value and elongation value.

**Figure 14:**
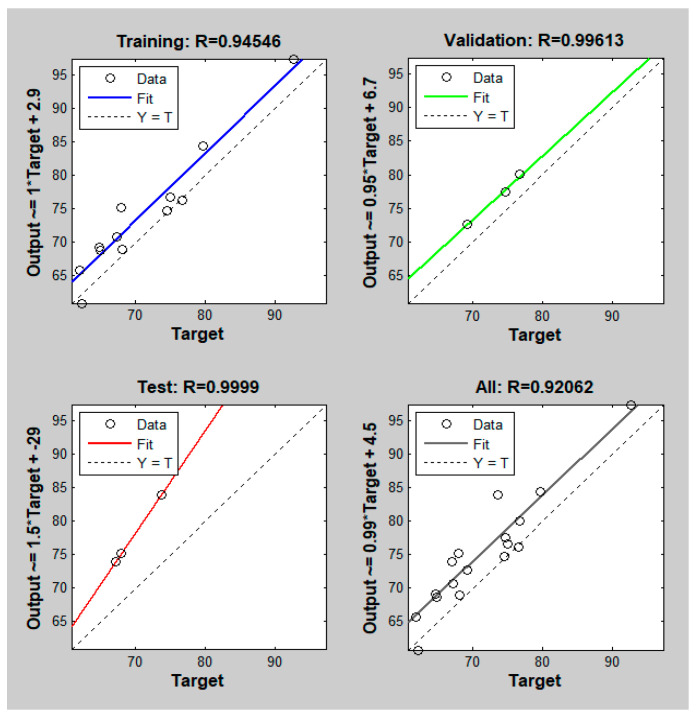
The R-squared results of PA6 + 30% FG composite samples with weld lines.

Separately from the comparisons in Table 2 with traditional observation, we also try to optimize the parameters by another tool. We conduct an optimization method called ANNs. ANNs is a computer program motivated by biology that imitate how the human brain processes information. ANNs design is trained by finding patterns and correlations in data and learning through experience rather than programming. An ANN is made consisting of hundreds of single units, artificial neurons, or processing elements, which are linked together using coefficients to form the neural structure and are structured in layers. We use Matlab R2014a, the neural network design comprises five input nodes, ten hidden neurons, and one output node. First, the neural network is trained using the input and output parameters. Figure 14 shows the R-squared results of PA6 + 30% FG composite samples with a weld line. When the R-squared result is near to 1 or −1, it is regarded as reasonable. The correlation between variables in a regression model is measured by R-squared. It shows the degree of agreement between predicted and actual values. According to the graph above, a value near to 1 implies a high positive correlation between the variables. This suggests that the model predicts well and can explain a substantial portion of the variability in the actual data. The prediction results after training produce an optimal performance level for the given training parameters.

The network output and experimental testing data for PA6 30% GF are compared in Table 3. It can be seen that the predicted results of ANN mostly agree well with the experimental data, while the maximum relative deviation is only 8.4%.

After training the neural network for the input and output data, we proceed with GA (Genetic Algorithm) to optimize the results. The purpose is to find the best parameters that generate the best outcome. We use the gamultiobj algorithm tool to perform GA for 5 input variables corresponding to five input parameters: filling time, packing time, packing pressure, melt temperature, and mold temperature. In this case, the lower bound is [3 0 59 265 30], and the upper bound is [3.8 0.8 63 273 173]. Table 4 shows that the expected strength result is 85.1 MPa, corresponding to the optimal parameters of filling time 3.0 s, packing time 0.312 s, packing pressure 55.45 MPa, melt temperature 265.74 °C, and mold temperature 97.7 °C. This ANNs-supported 85.1 MPa result is 15% greater than the highest UTS value of sample 4, indicating the possibility of improving the material strength by further optimization process.

### 3.2. SEM Analysis

**Figure 15:**
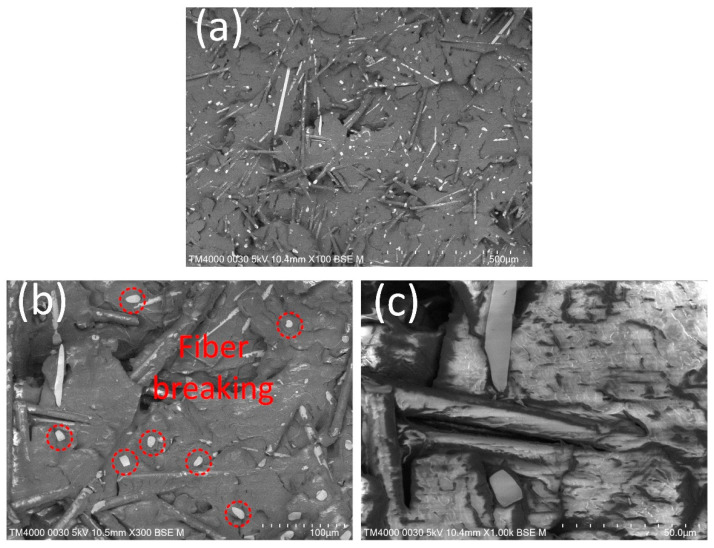
SEM picture of the fracture surface of PA6 + 30% FG composite sample at different magnifications: (**a**) ×100, (**b**) ×300, and (**c**) ×1000.

The fracture surfaces of the PA6 + 30% FG composite sample at different magnifications are displayed in Figure 15. There is fiberglass scattering on the PA matrix. The average fiberglass length is 237 µm, and the average fiberglass diameter is 11 µm. The boundary between the fiberglass and the PA matrix indicates a good adhesion between them. During the tensile process, cracks form at the ends of fibers. Then, cracks propagate through the matrix along the interface. Finally, matrix cracks emerge from interfacial cracks, possibly due to matrix plastic deformation [46]. Notably, the fracture surface is smooth, pointing out the high brittle level of the PA6 + 30% FG composite sample, as shown in Figure 15a. The low elongation values, ranging from 4.30% to 4.80%, also prove the brittleness of the weld line area. Many pieces of fiberglass are broken in the fracture surface, indicating the bridging of the fiber through the weld line area, as shown in Figure 15b. This fiber bridging mechanism could increase the strength of the weld line [28]. Therefore, increasing the weld line strength by adding fiberglass has good efficiency due to the bridging mechanism of the fiber through the weld line interface.

## 4. Conclusions

This study investigates the impact of injection parameters on the weld line strength of the PA6 + 30% FG composite samples. The effects of filling time, packing time, packing pressure, melt temperature, and mold temperature on the tensile test result of the weld line are examined. Some significant findings might be withdrawn, including:

The injection parameters of 3.6 s of filling time, 0.4 s of packing time, 59 MPa of packing pressure, and 269 °C of melt temperature result in the maximum UTS value of 74 MPa. In addition, the highest elongation value of 4.98% is obtained by the injection parameters of filling time of 3.4 s, packing time of 0 s, packing pressure of 59 MPa, and melt temperature of 269 °C.

Compared to other factors, the filling time factor has the lowest impact rate. On the contrary, the packing pressure has the most considerable UTS standard deviation value, indicating that this factor significantly affecting the UTS value.

The melt temperature factor has the highest elongation standard deviation of 0.39%, pointing out the strong impact of melt temperature on the elongation value. In reverse, the filling time factor has the lowest elongation standard deviation of 0.14%, showing the low result of this factor on the elongation value.

The packing pressure factor needs to be carefully taken into account in order to increase the UTS value of the PA6 composite. When taking into account the elongation value, the melt temperature factor must be taken into account.

The elongation value is greatly improved by raising the mold temperature because a higher temperature creates a better connection in the weld line area. When using the mold temperature control system, the UTS value slightly improves. The mold temperature parameter results in better elongation than expected. While the UTS result from all factors shows a little deviation, it is lower than expected.

The ANNs result with Genetic Algorithm optimization shows that the optimal strength result is 85.1 MPa, corresponding to the optimal parameters of filling time 3.0 s, packing time 0.312 s, packing pressure 55.45 MPa, melt temperature 265.74 °C, and mold temperature 97.7 °C.

The SEM results demonstrate that the boundary between the fiberglass and the PA matrix has high adhesion. The fracture surface is smooth, indicating that the PA6 + 30% FG composite sample has a high fragility level. Increasing weld line strength by adding fiberglass has a high efficiency due to the fiber’s bridging mechanism at the weld line interface.

According to the analysis, the packing pressure and melt temperature factors have the biggest impact on the mechanical characteristics of the weld line area. It also shows how well the mold temperature control system is working to increase the strength of the weld lines in the injection sample. These findings might help improve the quality of the injection molding sample with the weld line. For instance, the mechanical properties of the injection samples could be effectively improved by modifying the packing pressure, and melt temperature rather than filling time, and packing time.

## Figures and Tables

**Table 1:** Molding conditions for PA6 + 30% FG samples.

No.	Filling Time (s)	Packing Time (s)	Packing Pressure (MPa)	Melt Temperature (°C)
1	3.0	0.4	59	269
2	3.2			
3	3.4			
4	3.6			
5	3.8			
6	3.4	0	59	269
7		0.2		
8		0.4		
9		0.6		
10		0.8		
11	3.4	0.4	55	269
12			57	
13			59	
14			61	
15			63	
16	3.4	0.4	59	265
17				267
18				269
19				271
20				273

**Table 2:** Average UTS and elongation values with standard deviations of the PA6 + 30% FG composite samples with weld lines at different injection factors.

Factors	Average UTS (MPa)	UTS Standard Deviation (MPa)	Average Elongation (%)	Elongation Standard Deviation (%)
Filling time	73.36	0.70	4.65	0.14
Packing time	70.80	1.54	4.80	0.19
Packing pressure	70.94	2.33	4.51	0.23
Melt temperature	70.82	2.08	4.30	0.39

**Table 3:** Comparison between the network output and experimental testing data for PA6 + 30% GF.

No.	Experiment (MPa)	Network Output (MPa)	Relative Deviation (%)
1	73.6	74.1	0.7
2	72.1	73.8	2.4
3	73.4	72.5	1.2
4	74.0	73.1	1.2
5	73.7	73.8	0.1
6	71.3	72.6	1.8
7	69.7	70.8	1.6
8	73.4	70.5	4.0
9	68.9	74.7	8.4
10	70.6	73.4	4.0
11	73.2	72.5	1.0
12	71.3	68.4	4.1
13	73.4	67.3	8.3
14	67.2	71.6	6.5
15	69.6	73.5	5.6
16	69.7	72.5	4.0
17	67.4	71.1	5.5
18	73.4	72.1	1.8
19	71.9	76.8	6.8
20	71.7	74.3	3.6

**Table 4:** The Pareto table of optimized results for PA6 + 30% GF.

PA6 30% GF	Filling Time (s)	Packing Time (s)	Packing Pressure (MPa)	Melt Temperature (°C)	Mold Temperature (°C)
ANN + GA	3.0	0.312	55.45	265.74	97.74

## Data Availability

The data used to support the findings of this study are available from the corresponding author upon request.
